# The Effect of Glucocorticoid and Glucocorticoid Receptor Interactions on Brain, Spinal Cord, and Glial Cell Plasticity

**DOI:** 10.1155/2017/8640970

**Published:** 2017-08-08

**Authors:** Kathryn M. Madalena, Jessica K. Lerch

**Affiliations:** ^1^Department of Neuroscience, The Ohio State University, Columbus, OH 43210, USA; ^2^Center for Brain and Spinal Cord Repair, The Ohio State University, Columbus, OH 43210, USA

## Abstract

Stress, injury, and disease trigger glucocorticoid (GC) elevation. Elevated GCs bind to the ubiquitously expressed glucocorticoid receptor (GR). While GRs are in every cell in the nervous system, the expression level varies, suggesting that diverse cell types react differently to GR activation. Stress/GCs induce structural plasticity in neurons, Schwann cells, microglia, oligodendrocytes, and astrocytes as well as affect neurotransmission by changing the release and reuptake of glutamate. While general nervous system plasticity is essential for adaptation and learning and memory, stress-induced plasticity is often maladaptive and contributes to neuropsychiatric disorders and neuropathic pain. In this brief review, we describe the evidence that stress/GCs activate GR to promote cell type-specific changes in cellular plasticity throughout the nervous system.

## 1. Glucocorticoids and Actions of the Glucocorticoid Receptor

GCs function to maintain homeostasis and are released in response to stress, to reduce inflammation, and to combat low blood sugar [1]. Once in the bloodstream, GCs diffuse into tissues and cells and bind to the widely expressed glucocorticoid (GR) or mineralocorticoid nuclear receptors (MR). At basal levels, GC binding favors MRs; however, GRs are still occupied by GCs at low levels [2]. When GCs become elevated (e.g., with stress), GCs bind GRs at higher levels than baseline. Unbound GR is in the cytoplasm in a complex with heat shock proteins, HSP90 and HSP70, and FK506-binding proteins, FKBP51 and 52, and, upon binding GC, undergoes a conformational change and nuclear translocation to mediate gene transcription [3–5]. GRs activate gene transcription by binding to glucocorticoid response elements (GREs) or through direct protein-protein interactions (e.g., with AP1 transcription factors) [1, 6]. GR can also form a heterodimer with MR, giving this complex the ability to affect gene transcription in a way unique to both GR and MR [7]. GR binding to negative GREs (nGREs) can silence gene expression by competing with and displacing other transcription factors [1]. GR can also signal through nongenomic pathways which occur rapidly and do not require transcriptional changes [1, 8]. This nongenomic signaling occurs when the activity of kinases is altered upon ligand binding to GR [1, 8]. A prominent example is a reduction in the proinflammatory molecule arachidonic acid which occurs after release of c-Src from unliganded GR [1, 8].

Numerous transcriptional GR targets inhibit cytokine release (e.g., TNF*α* and IL-1*β*) making GR activation a powerful way to resolve inflammation [1, 9]. For this reason, GCs are commonly prescribed to treat acute and chronic inflammatory disease, autoimmune disease, and after organ transplant [3]. Unfortunately, their therapeutic benefits are limited by side effects. Less severe side effects include the return of inflammation or pain to the administration site, while more severe side effects of chronic use include osteoporosis, abdominal obesity, glaucoma, growth retardation in children, and hypertension [3, 8]. Additionally, GC administration can result in neuropsychiatric disorders such as affective, behavioral, and cognitive syndromes like depression, mood imbalance, and anxiety [10]. Clearly, the actions of GC straddle the line between adaptive and maladaptive and are not entirely understood.

## 2. Glucocorticoid and Mineralocorticoid Receptor Expression in the Nervous System

While GR is ubiquitously expressed, GR expression levels are not equal. GR expression in sensory neurons can vary depending on the sensory neuron subtype [11]. Calcitonin gene-related peptide-positive (CGRP+) peptidergic, isolectin B4-positive (IB4+) nonpeptidergic, proprioceptive, and mechanoreceptive neurons express GR, but IB4+ neurons have the highest expression [11]. In the central nervous system (CNS), neurons throughout the brain express GR, but hippocampal CA1 neurons have the highest level of expression [12, 13]. In addition to neurons, all glia express GRs. Schwann cells, the myelinating cells of the peripheral nervous system; brain and spinal cord microglia, which originate from bone marrow-derived monocytes and are the resident macrophages of the CNS; oligodendrocytes, the myelinating cells of the CNS; and astrocytes, which are considered the major support cells to CNS neurons, all bind synthetic GR agonists and are positive for GR immunoreactivity [11, 14–16]. GR mRNA is the most abundant steroid receptor found in Schwann cells, with an approximately 10-fold enrichment over MR transcript [17]. In brain microglia GR, mRNA levels are greater than both MR and estrogen receptors [15], suggesting that microglia could be uniquely sensitive to GC-GR interactions. GRs likely affect astrocyte and oligodendrocyte differentiation since GRs are found in O2A progenitor cells [16], and GCs affect their differentiation [18]. Once differentiated, GR levels in astrocytes are greater than those in oligodendrocytes and their precursors, which suggest that astrocytes are more sensitive to GC-GR interaction than oligodendrocytes [16]. GR expression in oligodendrocytes, while lower than that in astrocytes, can be clearly detected in the corpus callosum and white matter in the hippocampus [19]. The relative overabundance of GRs found in glia involved in inflammatory processes (e.g., microglia and astrocytes) suggests that they respond more to GC-GR interactions than myelinating glial cells (e.g., oligodendrocytes and Schwann cells). This observation is not surprising given the distinct roles of GRs in inflammatory modulation [20].

GR can autoregulate its expression, and prolonged GC exposure induces GR downregulation [21]. This downregulation might be stress-type dependent more than GC level dependent. Neither chronic intermittent stress for 30 days nor three days of high-dose corticosterone resulted in reduced GR transcription in rats, despite elevated levels of circulating GCs [22]. There is evidence for basal regulation of GR expression because adrenalectomy increases GR mRNA and protein in the hippocampus [22]. GR can clearly regulate its expression. Differences in autoregulation could be due to its interactions with GCs or other steroid receptors (e.g., MR).

MRs are expressed in neurons and glia, but expression can vary depending on cell type [23]. MRs colocalize with CGRP + peptidergic nociceptive neurons in the spinal cord and dorsal root ganglia [23]. In contrast, oligodendrocytes, spinal cord astrocytes, and microglia are nearly absent of MR immunoreactivity [19, 23]. It is likely that these cell types that lack MR but have high GR (e.g., spinal astrocytes and microglia) [11, 23] are more sensitive to GCs due to the absence of competitor receptor binding. Because glial cells become activated and undergo hypertrophy in response to injury and GCs, processes central to the development of pain and psychiatric disorders (e.g., anxiety-like behavior) [24–27], their unique GC sensitivity could indicate that GR activation and downstream pathways directly contribute to the exacerbation of these conditions. Indeed, stress/GCs transiently increase spinal cord microglia activation perhaps by increasing their activation rate [28], providing evidence for direct stress/GC-induced plasticity in microglia.

## 3. Neuronal Plasticity Induced by Glucocorticoids

Plasticity in the nervous system is defined by structural and physiological changes caused by increased or decreased neuronal activity or an endogenous ligand (e.g., GCs and neurotransmitters). Given GR's widespread neural expression, it is not surprising that increasing the amount of GC can induce structural plasticity in the CNS. These studies are frequently performed in rodents after exposure to a stressor (e.g., restraint, tail shock, immobilization, maternal separation, and social defeat). The consequence of stress on neural plasticity is widely varied. Sustained daily immobilization lasting at least one week induces a prolonged and continuous GC elevation, causes hippocampal CA3 pyramidal neuron apical dendritic atrophy [29], and expands dendritic arborization and length in basolateral amygdala (BLA) neurons (Table 1) [30]. This contrasting pattern of morphological plasticity between brain regions may be due to differences in synaptic plasticity [30]. In fact, chronic restraint alters the postsynaptic densities (PSDs) of thorny excrescences receiving input from mossy fiber boutons [31]. 3D reconstruction revealed a reduced number of PSDs per thorny excrescence, decreased volume of thorny excrescences, and a reduction in the surface area occupied by thorny excrescences [31]. Chronic restraint stress also induces changes in the CA1 region of the hippocampus. An early study showed that chronic restraint reduced the volume of granule and pyramidal CA1 neurons, and these changes are associated with deficits in learning and memory [32]. There was slight recovery one month after stress, suggesting the effects of stress are not permanent [32]. A second study showed a reduction in dorsal anterior CA1 and alterations in synapse connectivity in CA1 axospinous synapses, specifically an increase in the size of PSDs [33]. Chronic restraint also decreased the dendritic length and spine density of apical, but not basilar dendrites in medial prefrontal cortical neurons (Table 1) [34]. This plasticity is dependent on the N-methyl-D-aspartate (NMDA) receptor and is reversed by a NMDA receptor antagonist which even produces the opposite effect (e.g., hypertrophy of apical dendrites) [35]. Chronic unpredictable stress (CUS) in rats disrupts hippocampus-to-prefrontal cortex (PFC) synapses, reduces prefrontal layers I and II volume, and is correlated with working memory and behavioral flexibility problems [36]. The PFC is important in these behavior tasks, and it is clear that chronic stress disrupts the normal function of the PFC.

Stress/GC exposure can have profound effects on emotion and cognition, specifically, and can increase fear conditioning in rats after chronic restraint stress [37]. The polysialylated neuronal cell adhesion molecule is a marker of developing neurons but remains in adulthood in brain regions capable of morphological plasticity [38]. Chronic restraint stress reduces this molecule in the hippocampus and amygdala while increasing fear conditioning [37, 39]. These studies provide evidence that this change in behavior is caused by a stress-induced reduction in this neuromodulatory molecule in areas critical to fear development (e.g., amygdala). Other stress-induced behavioral changes include decreased sociability and increased aggression [40]. This behavioral change in chronically restrained rats was attributed to a decrease in neuroligin 2 in the hippocampus [40]. Neuroligins play a role in proper synapse formation function [40] which provides further evidence that stress changes neuromodulatory molecules in critical brain regions, leading to changes in emotion and cognition [37, 39]. There is also electrophysiological evidence for disruption of brain circuitry after stress, leading to cognitive disturbance. Rats subjected to CUS had impaired spatial reference memory which was attributed to a decreased coherence between ventral hippocampus and medial PFC [41]. Coherence is the matching of the temporal structure of signals between brain regions, which are synchronous if the phase matches [41]. Reduced coherence between regions provides evidence for disrupted connections. In this study, there was increased signal amplitude in the ventral hippocampus which is thought to compensate for the disruption in this circuit [41]. Stress/GCs can increase or decrease glutamate release, depending on the type of stressor. For example, hippocampal glutamate levels significantly increase after acute immobilization stress, peaking twenty minutes after stress onset and again immediately after its conclusion [42]. Adrenalectomy reversed this increase as well as reduced basal glutamate levels, suggesting that GCs regulate glutamate release both after stress and at baseline [42]. Another study found that acute restraint stress increases hippocampal, septal, and frontal cortex glutamate release and reuptake [43]. Combining restraint stress with tail shock induces the opposite effect, reducing glutamate reuptake in the CA1 region of the hippocampus and increasing long-term depression (LTD) [44]. The GR antagonist RU38486 reverses this effect suggesting that GC-GR interactions are upstream of synaptic machinery that modulates glutamate reuptake and produces LTD [44]. Interestingly, there is increasing evidence that the same stressor can elicit brain area-specific changes in glutamate release; tail pinch stress sharply increases PFC glutamate release and also in the hippocampus but to a lesser degree [45]. These data indicate that the downstream effects from GC-GR activation can be specific to neuronal populations and possibly individual neurons.

In addition to NMDA receptor function and glutamate release, stress/GCs can modulate neurotransmission by affecting the readily releasable pool of vesicles [46]. PFC neuron SNARE proteins increase after acute footshock, an effect that is reversed by the GR antagonist, RU486, suggesting that SNARE protein recruitment to the presynaptic membrane is downstream of GR activation [46]. While the above data clearly demonstrate that stress modulates acute neurotransmission in rodents, the effects of sustained GC level or chronic stress on neurotransmission are less studied. Chronic restraint stress reduces glutamate uptake in spinal synaptosomes in vitro [47]. In vivo, chronic restraint increased unstimulated glutamate release in cerebrocortical synaptosomes [48]. These studies provide evidence that chronic stress promotes changes in synaptosomal glutamate release and uptake in the CNS.

Acute and chronic stress stimuli differ in GC exposure length (e.g., hours to days to weeks), and investigations into whether this differentially modulates neurotransmission are ongoing. There is evidence that acute increases and chronic stress decrease synaptic glutamate reuptake and clearance [22, 23, 27, 28]. Forty days of random stressors decreased hippocampal glutamate uptake and potentially increased glutamate release [49]. Reduced uptake and increased release could potentially produce long-lasting changes in synaptic plasticity.

Chronic stressors affect neuronal plasticity, but the outcome changes depending on GC level and length of GC elevation. CUS induces GC elevation to a lesser extent than chronic immobilization, and subsequently, hippocampal CA3 dendrites remain unaffected, while retracting after chronic immobilization [30]. Interestingly, CUS causes the opposite effect in BLA neurons (e.g., retraction in dendritic length) compared to the hypertrophy observed after chronic immobilization (Table 1) [30]. These data indicate that neuronal cells respond differently according to the amount and length of time GCs are elevated. Chronic immobilization induces greater adrenal hypertrophy compared to CUS which inversely correlates with the amount of dendritic atrophy [30], demonstrating that higher GC levels cause considerable changes in CNS plasticity. A study on tree shrews found that chronic psychosocial stress decreased subthreshold excitability in hippocampal CA3 pyramidal neurons but did not change active membrane properties [50]. Chronic psychosocial stress was associated with decreased dendritic arborization (Table 1) [50], suggesting that all variations in the structural organization have real consequences on neuronal excitability and function. These data also suggest that the effects of stress/GCs on plasticity are not always repressing growth and are cell- and stress-type dependent.

GC-induced plasticity is not limited to the stress paradigms described above. Early life stress (e.g., maternal separation) causes basal dendritic tree atrophy, reduces spine density on second-order apical and basal dendritic branches in pyramidal neurons (Table 1), and impairs long-term potentiation [51]. This again provides evidence for diverse effects of different stress paradigms on neurons in the CNS.

The above studies all discuss changes induced by prolonged stress/GC elevation. Short bouts of stress/elevated GCs produce different effects on plasticity. Acutely elevated GC levels increase synaptic plasticity and facilitate hippocampal-dependent cognition while long-term exposure impairs synaptic plasticity and cognition, decreases neurogenesis and spine density, and causes dendritic atrophy [52]. Overall, acute rises in GCs tend to promote positive changes that support adaptation, while prolonged stress promotes negative adaptation evidenced not only by deleterious brain plasticity but also by severe side effects of long-term GC treatment discussed above [3, 8].

## 4. Glial Plasticity Induced by Glucocorticoids

Glial plasticity can be defined as changes in glial cell growth and function. While there are relatively few reports on the direct effects of GC-GR interaction in glial cells, we can infer GC-GR activity from the consequences of stress or GC administration from studies in animal models. For example, in culture, GC-GR interactions are the main components that drive Schwann cell proliferation [53], that contribute to myelination in neuron-Schwann cell cocultures [54], and that increase luciferase expression from the promoters of genes involved in Schwann cell myelination (e.g., peripheral myelin protein 2 and protein 0) [55]. While these are all in vitro studies, indirect in vivo evidence for GC-GR interactions on peripheral nerve myelination come from studies that use GCs to treat peripheral nerve injury. Indeed, GC treatment increases remyelination rate after injury (Table 1) [56] and could likely enhance axon regeneration, strongly suggesting a pivotal role for GC-GR interaction in Schwann cell function.

Microglial activation, which occurs in response to neuronal damage or soluble signals such as GCs, prompts microglia to change from their normal brain surveillance function to one associated with unfavorable effects on neural function and behavior [27, 28, 57–59]. Activated microglia undergo noticeable changes in morphology. Their long, thin processes turn into dense, broad protrusions [60]. These morphological changes are associated with an increase in inflammatory cytokine production and release (e.g., TNF*α*, IL-1*β*, CCL2, nitric oxide, and reactive oxygen species [59, 61]). Evidence that nervous system injury, stress, increased GCs, and even neurological disease are associated with and possibly directly contribute to microglial proliferation and activation (a form of plasticity) is growing. For example, after peripheral nerve injury, microglia become activated in the dorsal horn of the spinal cord and transiently increase in number (Table 1) [25, 26, 28, 62]. These changes are likely mediated at least in part through GC-GR-dependent mechanisms since microglia express low levels of MRs and other steroid hormone receptors [11, 14–16, 23], giving GCs the ability to signal directly to microglia. Stress/GCs and microglia interactions are also implicated in the onset and maintenance of psychological disorders. Repeated social defeat (a form of chronic psychosocial stress) changes brain microglia morphology and increases anxiety-like behavior in mice, an effect reversed by a *β*-adrenergic antagonist [27]. After repeated social defeat, the long, thin microglia process hypertrophy and become short and thick [27]. Microglial hypertrophy was observed in the medial amygdala, prefrontal cortex, hippocampus, and paraventricular nucleus (Table 1) [27]. While there is evidence for region-specific microglial cytokine release from specific types of stress [61], it would be interesting to examine if microglial activation and cytokine release are always region specific or system wide. Also, are microglia morphological changes and their activation occurring together? Overall, this is clear evidence that stress causes structural and functional changes in microglia and promotes maladaptive outcomes.

Endogenous GCs are long known for their ability to regulate oligodendrocyte differentiation and myelination. Early studies found that postnatal adrenalectomy profoundly increases myelination in the developing brain, which can be reversed by low-dose GC supplement [63]. In adult-adrenalectomized rats, GC administration strongly inhibits oligodendrocyte precursor cell proliferation (e.g., NG2 cells; Table 1) [64]. The profound effect that GCs have on oligodendrocyte myelination also comes from clinical studies that utilize synthetic GCs to treat white-matter disease such as multiple sclerosis (Table 1) [65]. These studies clearly indicate a role for GCs in oligodendrocyte myelination, but direct evidence for a dependence on GC-GR interactions is still lacking.

Astrocytes function in the tripartite synapse by being near the presynaptic and postsynaptic neurons where they clear synaptic glutamate to stop synaptic transmission [66]. Changes in glutamate clearance after stress/GC elevation suggest astrocytes could be involved [43, 49, 67]. There is evidence that chronic corticosterone exposure in male mice reduces astrocyte area in the hippocampus (Table 1) [68]. Chronic injection of corticosterone reduces hippocampal volume along with astrocyte number, somal size, and protrusion length (Table 1) and increases depressive-like behavior [68]. Chronic psychosocial stress in tree shrews also produced the same observations in astrocytes, but their behavior was not examined (Table 1) [69]. This reduction in astrocyte number and size could account for the reduced hippocampal volume and contribute to the depressive phenotype because decreased astrocyte volume occurred simultaneously to reduce hippocampal volume, a change that is associated with depression in humans [68]. Other studies provide evidence for stress-induced mental abnormalities, like the development of anxiety-like behavior [30], but mostly consider the neuronal plasticity as the cause. These recent findings on astrocyte plasticity [68, 69] present the idea that chronic GC elevation and GR activation are upstream of changes in astrocyte function.

## 5. GCs Promote Plasticity and Maintain Pain

GC-GR interactions are also being studied in the context of peripheral nerve injury. Both specific (dexamethasone 21-mesylate) and nonspecific (RU486) GR antagonism inhibit neuropathic pain after chronic constriction injury (CCI) [70, 71]. Also, adrenalectomy reduces, and synthetic GCs, such as dexamethasone, reestablish the neuropathic pain response [71], data suggesting that GC-GR interactions are critical for the establishment of neuropathic pain. Acute restraint stress (sixty minutes), before spared nerve injury (SNI), increases mechanical allodynia, an effect blocked by RU486 [28]. Administration of corticosterone to nonstressed mice induces allodynia similar to acute restraint stress [28]. These studies suggest that GC-GR interactions play a significant role in the generation of neuropathic pain, but the exact cell type and place of GC-GR interaction that is mediating this response needs further investigation.

NMDA receptors are also involved in GR-dependent neuropathic pain. There is a time-dependent upregulation of NMDA receptor subunits within the spinal cord dorsal horn after CCI which was significantly reduced by GR antagonism or an antisense oligonucleotide against GR [72]. Administration of the NMDA receptor antagonist memantine before acute restraint stress and SNI in mice lessened injury-induced allodynia, similarly to the GR antagonist [28]. These studies indicate that blocking GR activity or NMDA receptor signaling can also prevent GC-induced exacerbated hypersensitivity.

## 6. Conclusion

GC-GR interactions are responsible for diverse changes in the nervous system that are cell-type and stress-type specific. Prolonged GC exposure often leads to maladaptive neuronal and glial plasticity that consists of both structural and functional changes associated with, and perhaps underlying, the development of anxiety-like or neuropathic pain behavior [27, 28, 30, 70–72]. We suggest that astrocyte and microglial activation and hypertrophy are direct consequences of GC-GR activity given the high GR expression in these glial cells. These observations combined with stress/GCs' ability to affect synaptic function [42–45, 49, 67] and neurotransmission [46] place glia in a prime position to contribute to maladaptive behaviors. While the role for GC-GR interactions in myelinating glia is less clear, elevated GCs modulate myelination, suggesting that GR is influencing this process. The structural and synaptic plasticity associated with GC-GR interactions in cell types throughout the nervous system implies that these changes have a real physiological relevance in the development of different disorders and that GC-GR interactions have a widespread function beyond modulating cellular growth.

## Figures and Tables

**Table 1:** Morphological plasticity after GC-GR interaction varies with stress and cell type. The paucity of studies in astrocytes and microglia highlights the need for additional studies.

Stress type	Duration	Species	Sex	Neurons	Schwann cells	Microglia	Oligodendrocytes	Astrocytes
Immobilization	Chronic	Rat	♂	Atrophy, CA3 hippocampus [29, 30]	—	—	—	—
				Hypertrophy, basolateral amygdala [30]				
Restraint	Chronic	Rat	♂	Decreased dendritic length and spine density, medial prefrontal cortex [34]	—	—	—	—
Unpredictable stress	Chronic	Rat	♂	Decreased dendritic length, basolateral amygdala [30]	—	—	—	—
Psychosocial stress	Chronic	Tree shrew	♂	Decreased dendritic arborization, CA3 hippocampus [50]	—	—	—	Reduced astrocyte area, number, somal volume, protrusion length, hippocampus [69]
Maternal separation	Chronic	Rat	♂	Atrophy, basal dendritic tree, reduced spine density, apical & basal dendrites layer II/III pyramidal neurons [51]	—	—	—	—
Corticosterone injection	Chronic	Mouse	♂	—	Increased rate of myelination, sciatic nerve [56]	—	—	Reduced astrocyte area, number, somal volume, protrusion length, hippocampus [68]
		Rat			—		Decreased proliferation of NG2 cells [64]	—
Restraint + spared nerve injury	Acute	Mouse	♀	—	—	Increased reactivity, dorsal horn [28]	—	—
Repeated social defeat	Chronic	Mouse	♂	—	—	Hypertrophy, medial amygdala, prefrontal cortex, hippocampus, paraventricular nucleus [27]	—	—
Synthetic glucocorticoid injection	Chronic	Human	♂/♀	—	—	—	Increased CNS myelination [65]	—
